# Anti-Cancer Properties of the Naturally Occurring Aphrodisiacs: Icariin and Its Derivatives

**DOI:** 10.3389/fphar.2016.00191

**Published:** 2016-06-29

**Authors:** Hui-Li Tan, Kok-Gan Chan, Priyia Pusparajah, Surasak Saokaew, Acharaporn Duangjai, Learn-Han Lee, Bey-Hing Goh

**Affiliations:** ^1^Novel Bacteria and Drug Discovery Research Group, School of Pharmacy, Monash University MalaysiaBandar Sunway, Malaysia; ^2^Biomedical Research Laboratory, Jeffrey Cheah School of Medicine and Health Sciences, Monash University MalaysiaBandar Sunway, Malaysia; ^3^Division of Genetic and Molecular Biology, Faculty of Science, Institute of Biological Sciences, University of MalayaKuala Lumpur, Malaysia; ^4^Center of Health Outcomes Research and Therapeutic Safety (Cohorts), School of Pharmaceutical Sciences, University of PhayaoPhayao, Thailand; ^5^Pharmaceutical Outcomes Research Center, Faculty of Pharmaceutical Sciences, Naresuan UniversityPhitsanulok, Thailand; ^6^Division of Physiology, School of Medical Sciences, University of PhayaoPhayao, Thailand

**Keywords:** icariin, icaritin, icariside II, Yin Yang Huo, anti-cancer, ethnopharmacology

## Abstract

*Epimedium* (family Berberidaceae), commonly known as Horny Goat Weed or Yin Yang Huo, is commonly used as a tonic, aphrodisiac, anti-rheumatic and anti-cancer agent in traditional herbal formulations in Asian countries such as China, Japan, and Korea. The major bioactive compounds present within this plant include icariin, icaritin and icariside II. Although it is best known for its aphrodisiac properties, scientific and pharmacological studies suggest it possesses broad therapeutic capabilities, especially for enhancing reproductive function and osteoprotective, neuroprotective, cardioprotective, anti-inflammatory and immunoprotective effects. In recent years, there has been great interest in scientific investigation of the purported anti-cancer properties of icariin and its derivatives. Data from *in vitro* and *in vivo* studies suggests these compounds demonstrate anti-cancer activity against a wide range of cancer cells which occurs through various mechanisms such as apoptosis, cell cycle modulation, anti-angiogenesis, anti-metastasis and immunomodulation. Of note, they are efficient at targeting cancer stem cells and drug-resistant cancer cells. These are highly desirable properties to be emulated in the development of novel anti-cancer drugs in combatting the emergence of drug resistance and overcoming the limited efficacy of current standard treatment. This review aims to summarize the anti-cancer mechanisms of icariin and its derivatives with reference to the published literature. The currently utilized applications of icariin and its derivatives in cancer treatment are explored with reference to existing patents. Based on the data compiled, icariin and its derivatives are shown to be compounds with tremendous potential for the development of new anti-cancer drugs.

## Introduction

Cancer is a growing health problem, representing one of the leading causes of mortality worldwide (Goh and Kadir, 2011). The current standard of care includes conventional medicine, surgery, radiotherapy, and chemotherapy (Hsiao and Liu, 2010) however the limitations of existing care are clear as even after completion of the standard treatment regimens, there are high rates of metastasis and relapse (Liang et al., 2010). In addition, there are significant problems associated with the standard treatments such as significant toxicity levels and the emergence of resistance toward anti-cancer drugs, making treatment of cancer even more challenging (Crespo-Ortiz and Wei, 2012). Consequently, the annual death rates due to cancer cannot be significantly lowered (Chan et al., 2015) unless new treatment strategies can be devised. Scientific exploration of medicinal plants and herbs is now of increasing interest in the hope that they will yield novel compounds with properties that can overcome the limitations of the drugs currently available (Lau et al., 2015; Supriady et al., 2015). Plant based compounds are also of interest due to their multitargeting properties, low cost, relative absence of toxicity and high availability (Gupta et al., 2011; Tan et al., 2015; Sayyad et al., in press).

*Epimedium* (family Berberidaceae), (Latin name *Herba Epimedii*) commonly known as Horny Goat Weed or Yin Yang Huo in Chinese, is a genus consisting of approximately 52 species of herbaceous flowering plants (Ma et al., 2011). In recent years, there has been a significant volume of research on the anti-cancer effect of the main active compounds from *Herba Epimedii* such as icariin, icaritin, and icariside II. Icariin and its derivatives, icaritin, and icariside II seem to be promising compounds for cancer treatment, with studies having shown that they exhibit anti-cancer activity against a wide range of cancer cell types such as osteosarcoma (Geng et al., 2014), prostate (Lee et al., 2009), lung (Zheng et al., 2014), gastric (Wang et al., 2010), and kidney cancer cells (Li et al., 2013b). These compounds exert their anticancer action via a multitude of cellular targets and through a variety of pathways including apoptosis inducing effect, cell-cycle modulation, anti-angiogenesis, anti-metastasis, and immunomodulation. Of particular interest, they effectively target cancer stem cells and drug resistant cancer cells. Research also suggests they are able to potentiate the current standard cancer treatments. The purpose of this review is to provide an up-to-date of the anti-cancer mechanisms of icariin and its derivatives; and to provide scientific evidence that there is a basis to support the efficacy of *Herba Epimedii*-based traditional herbal formulations for the treatment of cancer. Overall, the existing literature highlights the remarkable potential of these compounds for the development of novel drugs for cancer treatment.

## Plant description and traditional uses

*Epimedium* is a low-growing, deciduous plant with leathery leaves that spreads by underground stems. The flowers of *Epimedium* vary in color and they have eight sepals. A tough and long-lived perennial species, *Epimedium* is found mainly on cliffs in moist forests, near streams and wet lands at altitudes of between 200 and 3700 m (Ma et al., 2011). *Epimedium* is widely distributed from Japan to Algeria but it is mostly found in the East Asian and Mediterranean region (Arief et al., 2015).

*Epimedium* species have a long history of use in traditional medicine as they have been used in botanical supplements for more than 2000 years. The extracts of *Epimedium* plants are included in traditional herbal formulations for the treatment of infertility, rheumatism and cancer in Asian countries such as China, Japan, and Korea. In China, *Epimedium* is taken as a supplement for prevention of chronic diseases and to strengthen the body (Cassileth et al., 2010; Ma et al., 2011). Today, *Herba Epimedii* is still popular in the treatment of cancers; it has been commonly used as one of the main ingredients together with other herbs, for the preparation of traditional Chinese formulations to treat various cancers such as digestive system cancers, hepatocarcinomas, lung cancers, breast cancers and cervical cancers (Zhang, 1991; Qi and Qi, 2002; Wang, 2003; Teng, 2010). With reference to formal research, the extracts of *Herba Epimedii* were reported to display anti-cancer activity in cancer cell lines such as colon cancer cells, hepatoma and leukemia cells (Lin et al., 1999; Guon and Chung, 2014). Given the apparent efficacy of *Herba Epimedii* in treating cancers, phytochemical analyses have also been carried out to identify the bioactive components responsible for its pharmacological activities.

## Icariin and its derivatives

**Figure 1:**
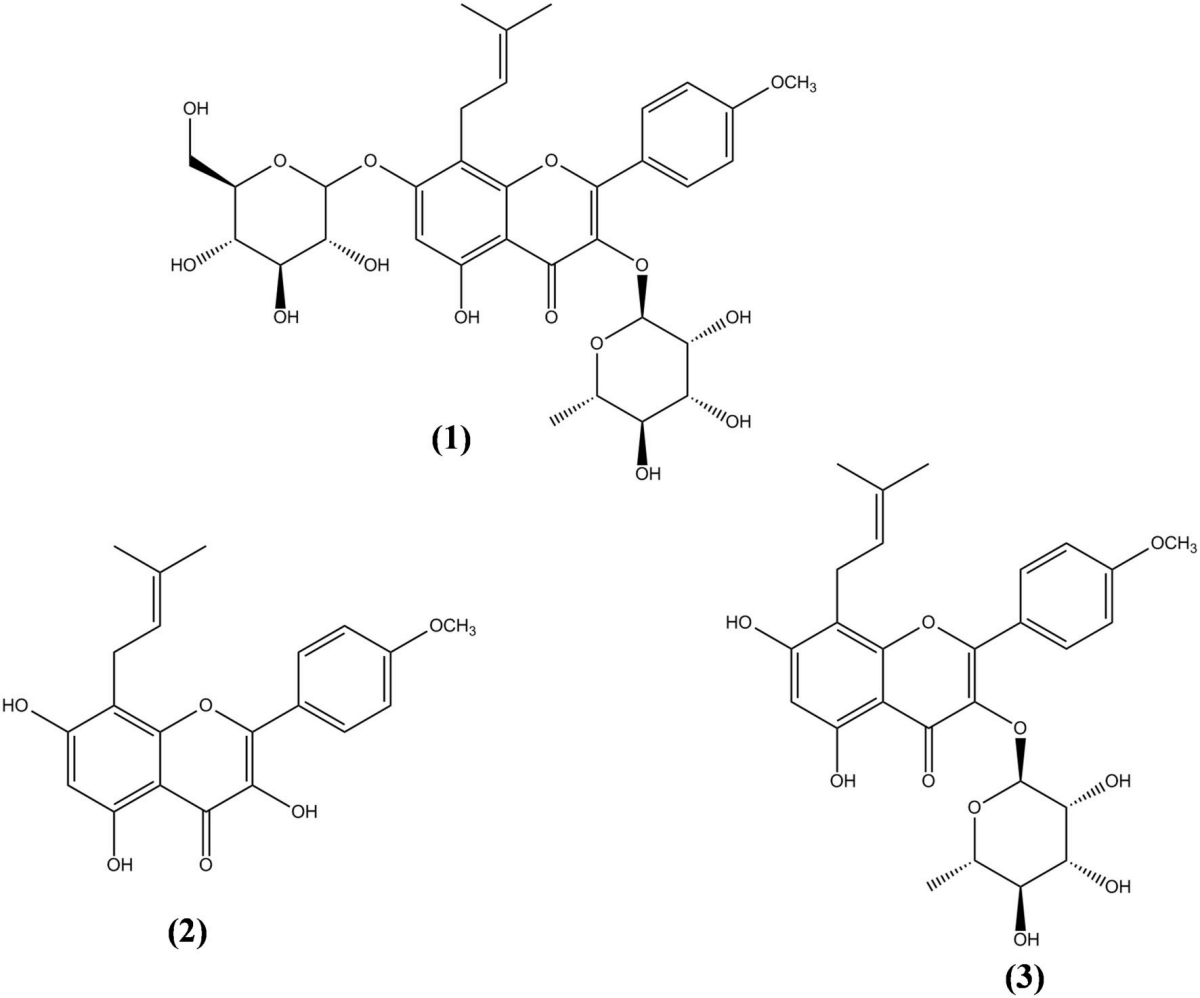
**Chemical structures of icariin and its derivatives isolated from ***Herba Epimedii*****. Icariin (1), icaritin (2), and icariside II (3).

More than 260 moieties can be detected in *Herba Epimedii*, including flavonoids, lignins, ionones, phenol glycosides, phenylethanoid glycosides, sesquiterpenes, and other types of compounds (Ma et al., 2011). Among the compounds identified, icariin is thought to be the principal bioactive compound in *Epimedium* extracts. Aside from icariin, derivatives such as icaritin, icariside I, icariside II, and desmethylicaritin can also be found in *Herba Epimedii* (Ma et al., 2011). Metabolic and pharmacokinetic studies have shown that these derivatives can also be obtained through the metabolism of icariin by intestinal flora, by converting icariin to icaritin, icariside I, icariside II, and desmethylicaritin (Liu et al., 2005; Xu et al., 2007). As shown in Figure 1, icariin (1) is a prenylated flavonol glycoside with rhamnosyl, glucosyl, and methoxy groups. Deglycosylation or demethylation of icariin will form different metabolites. For instance, icariside I will be formed when the rhamnose residue is removed whereas icariside II (3) will be formed when the glucose residue is removed. Deglycosylation of icariin will result in icaritin (2) and demethylation of icaritin will result in desmethylicaritin (Shen et al., 2009; Khan et al., 2015).

Scientific studies have provided evidence of the broad therapeutic capabilities of icariin, especially with reference to its effects on reproductive function (Chen et al., 2014), osteoprotective (Qin et al., 2008), neuroprotective (Nie et al., 2010), cardioprotective (Ding et al., 2007), anti-inflammatory (Xu et al., 2010), and immunoprotective effects (Li et al., 2011). Icariin's derivatives have also been shown to possess significant biological activities. For example, icaritin was shown to possess osteopromotive (Yao et al., 2012), neuroprotective (Wang et al., 2007) and cardioprotective effect (Lei et al., 2015). The number of studies on icariside I are limited but it was reported to alleviate sexual dysfunction (Lenoble et al., 2002). Icariside II, is reported to possess anti-hepatotoxic (Cho et al., 1995), anti-osteoporosis effects (Liu et al., 2014) and the ability to ameliorate diabetic neuropathy (Tian et al., 2015). Icariin, icaritin and icariside II, have also been reported to possess anticancer activity. Results of both *in vitro* and *in vivo* experiments have suggested these compounds hold promise for cancer treatment because they are effective against a wide range of cancer cell types including osteosarcoma (Geng et al., 2014), prostate (Lee et al., 2009), lung (Zheng et al., 2014), gastric (Wang et al., 2010), kidney (Li et al., 2013b). Furthermore, these compounds seem to exert their anticancer actions through different cellular targets and pathways. It is crucial to attain a more complete understanding of the underlying mechanisms of the anticancer properties of icariin and its derivatives to provide direction for future research in cancer drug discovery.

## Anticancer properties

### Apoptotic activity

Over the past decade, triggering apoptosis in tumor cells has been utilized as an effective approach for treatment of cancer (Han et al., 2015). Apoptosis occurs through two different pathways: the extrinsic or death receptor pathway and the intrinsic or mitochondrial pathway (Goh et al., 2014). Both pathways of apoptosis involve the activation of initiator caspases, followed by activation of effector caspases (caspase-3, -6, and -7) which the major executioners of apoptosis (Mongiat et al., 2007; Ahmed and Othman, 2013). With reference to the intrinsic pathway, mitochondrial outer membrane permeabilization is the crucial event for initiator caspase activation (Chan et al., 2012). *Herba Epimedii* was shown to induce apoptosis of cancer cells; 70% ethyl alcohol extracts of *Epimedium koreanum* Nakai has caused activation of mitochondria-dependent apoptotic pathway in colon cancer cells (Guon and Chung, 2014). This led to further studies on the apoptotic activity induced by the bioactive components isolated from this herb. Generally, the apoptotic effect of icariin and its derivatives has been associated with the activation of the intrinsic pathway of apoptosis. Treatment with icariin and icaritin has resulted in increase in Bax/Bcl2 ratio, release of cytochrome c, cleavage of poly (ADP-ribose) polymerase and activation of caspases in a wide range of cancer cells (He et al., 2010; Li et al., 2010, 2013a, 2014b; Tong et al., 2011; Wang et al., 2011a; Zhu et al., 2011; Sun et al., 2015b; Wu et al., 2015b); these can be regulated by various signaling pathways, as shown in Table 1. Similarly, icariside II has been shown to increase the ratio of Bax/Bcl2, causing transposition of cytochrome c and activation of caspase-3 and -9 in cancer cells including lung cancer cells and acute myeloid leukemia cells. It also causes degradation of poly ADP-ribose polymerase (PARP) and reactive oxygen species (ROS) over-production (Kang et al., 2012; Song et al., 2012). Additionally, apoptosis of esophageal squamous cell carcinoma treated with icariside II has been reported to be associated with lowered expression of β-catenin and downregulation of expression of survivin and cyclin D1, suggesting that icariside II is capable of simultaneously inducing apoptosis while also inhibiting proliferation of cancer cells (Wang et al., 2011b). Other mechanisms involved include inhibition of epidermal growth factor receptor (EGFR) pathway activation (Wu et al., 2013b) which then prevents the downstream activation of the JAK/STAT3 pathway (Kim et al., 2011; Wu et al., 2013a). This shows the ability of icariside II to overcome the survival signals of tumor cells because STAT3 is one of the STAT proteins that is constitutively active in cancer cells where it promotes the expression of antiapoptotic proteins (Wu et al., 2013a). Icariside II also helps to overcome the survival signals of tumor cells by inhibiting cyclooxygenase-2 (COX-2)/PGE_2_ pathway (Lee et al., 2009); expression of COX-2 has been associated with tumor-cell resistance to apoptosis (Greenhough et al., 2009).

**Table 1:** **Apoptotic activity of icariin and its derivatives**.

**Compound**	**Cell Line**	**Study Type**	**Mechanism**	**References**
Icariin	Human lung adenocarcinoma cells (A459)	*In vitro* and *In vivo*	Activation of ERS signaling, increased expression of ERS-related molecules (p-ERK, ATF6, GRP78, p-eIF2α, and CHOP, downregulation of Bcl2 expression and upregulation of PUMA	Di et al., 2015
	Human esophageal cancer cells (EC109 and TE1)	*In vitro* and *In vivo*	Activation of ERS signaling, increased expression of ERS-related molecules (p-ERK, ATF4, GRP78, p-eIF2α, and CHOP, downregulation of Bcl2 expression and upregulation of PUMA	Fan et al., 2016
	Human hepatoma cells (SMMC-7721, Bel-7402, and HepG2)	*In vitro* and *In vivo*	Activation of ROS/JNK-dependent mitochondrial pathway that involved the generation of ROS and JNK activation, resulted in enhanced Bax-to-Bcl-2 ratio, loss of mitochondrial membrane potential, cytochrome c release, and caspase cascade	Li et al., 2010
	Mouse tumor Leydig cells (MLTC-1)	*In vitro*	Activation of caspase-9, -3, enhanced Bax/Bcl-2 ratio, and release of cytochrome c, as well as down-regulation of the expression of piwil4	Wang et al., 2011a
Icaritin	Hepatocellular Carcinoma (HepG2)	*In vitro*	Enhanced Bax/Bcl-2 ratio, activation of caspase-3, activation of JNK1 signaling	He et al., 2010
	Leukemia cells (Primary acute myeloid leukemia cells, NB4, HL60, and U937)	*In vitro*	Activation of caspase-9, -3, -7, cleavage of PARP, downregulation of c-myc, inhibition of MAPK/ERK, and PI3K/AKT signals.	Li et al., 2013a
	Human Burkitt lymphoma cells (Raji and P3HR-1)	*In vitro*	Activation of caspase-8, -9, cleavage of PARP, downregulation of c-myc and enhanced Bax/Bcl-2 ratio	Li et al., 2014b
	Human liver cells (L02) and Human hepatocarcinoma cells (SMMC-7721)	*In vitro*	Activation of caspase-3, -8, enhanced Bax/Bcl-2 ratio, increased expression levels of Fas	Sun et al., 2015b
	Human endometrial cancer cells (Hec1A)	*In vitro*	Activation of caspase-3 and caspase-9, cleavage of PARP, release of cytochrome c, enhanced Bax/Bcl-2 ratio, and sustained activation ERK1/2 signaling	Tong et al., 2011
	Extranodal NK/T-cell lymphoma cells (SNK-10 and SNT-8)	*In vitro*	Activation of caspase-3 and caspase-9, enhanced Bax/Bcl-2 ratio, downregulation of p-Bad, inhibition of Jak/Stat3, and PI3K/Akt signaling pathway mediated by reduced expression of LMP1	Wu et al., 2015b
	Leukemia cells (Primary chronic myeloid leukemia cells and K562)	*In vitro* and *In vivo*	Activation of caspase-3, -9, release of cytochrome c, enhanced Bax/Bcl-2 ratio, downregulated expression of Apaf-1, inhibition of MAPK/ERK/JNK signals and down-regulated kinase activity of Jak-2/STAT3/Akt signal network	Zhu et al., 2011
Icariside II	Human hepatoblastoma cells (HepG2), Mouse liver carcinoma cells (H22)	*In vitro* and *In vivo*	Mitochondrion membrane and lysomal membrane permeabilization, activation of caspase-3, -7, -8, -9, cleavage of PARP, enhanced Bax/Bcl-2 ratio.	Geng et al., 2015
	Human breast cancer cells (MCF-7)	*In vitro*	Activation of caspase-8, -9, decreased mitochondrial potential, release of cytochrome C and AIF, increased expression of Fas and FADD as well as increased expression of Bax and BimL	Huang et al., 2012
	Human acute myeloid leukemia cells (U937)	*In vitro*	Activation of caspase-3, cleavage of PARP, and decreased bcl-xL and survivin, inactivation of STAT3-related signaling pathway.	Kang et al., 2012
	Multiple myeloma cells (U266)	*In vitro*	Down-regulation of expression of Bcl-2, Bcl-xL, survivin, Cyclin D1, COX-2, and VEGF, enhanced PARP cleavage and caspase-3 activation, inhibition of STAT3 activation and enhanced expression of SHP-1 and PTEN through inhibiting JAK2 and c-Src	Kim et al., 2011
	Human prostate cancer cells (PC-3)	*In vitro*	Activation of caspase-8, -9, -3, cleavage of PARP, decreased mitochondrial potential, COX-2, iNOS, and VEGF expression, release of cytochrome C, inhibition of COX-2/PGE2 pathway	Lee et al., 2009
	Human non-small cell lung cancer cells (A549)	*In vitro*	Activation of caspase-3, -9, cleavage of PARP, release of cytochrome c, enhanced Bax/Bcl-2 ratio, Activation of ROS/MAPK pathway that involved activation of ROS downstream effectors, JNK and p38MAPK	Song et al., 2012
	Human eosophageal squamous carcinoma (Eca109)	*In vitro* and *In vivo*	Downregulation of survivin and Cyclin D1, inhibition of β-catenin-dependent signaling.	Wang et al., 2011b
	Human epidermoid carcinoma cells (A431)	*In vitro*	Activation of caspase-9, cleavage of PARP, inhibition of JAK/STAT3 and MAPK-ERK pathways, activation of PI3K/AKT pathway, inhibition of EGF-induced activation of EGFR pathway.	Wu et al., 2013b
	Human melanoma cells (A375 and SK-MEL-5) and Mouse melanoma cells (B16)	*In vitro* and *In vivo*	Activation of caspase-3, decreased expression of survivin, inhibition of JAK/STAT3 and MAPK pathways as well as activation of PI3K/AKT pathway	Wu et al., 2013a

The extrinsic pathway, on the other hand, is activated by specific receptors of the tumor necrosis factor receptor superfamily, such as Fas, DR4 (TRAIL-R1), and DR5 (TRAIL-R2). The binding of their specific ligands will result in the production of death-inducing signaling complex and activation of caspase-8 and caspase-10 which will then activate the effector caspases (Mongiat et al., 2007). In some studies, both intrinsic and extrinsic pathways seem to be involved in the induction of apoptosis by icariin and its derivatives. For example, the treatment of human hepatocellular carcinoma with icaritin not only triggered mitochondrial/caspase apoptotic pathway but also induced apoptosis through Fas-mediated caspase-dependent pathway as evidenced by increased levels of Fas and cleaved caspase-8 (Sun et al., 2015b). Similar results were obtained in breast cancer cells treated with Icariside II (Huang et al., 2012).

Aside from mitochondria, organelles such as endoplasmic reticulum and lysosomes also play an important role in apoptosis of cancer cells (Geng et al., 2015). The rapid rate of glucose metabolism and growth in cancer cells causes endoplasmic reticulum stress (ERS) and activation of the unfolded protein response (UPR). As the UPR is one of the resistance mechanisms against cancer therapy, it can often enable cancer cells to adapt to ERS and evade apoptotic pathways (Yadav et al., 2014). However, some studies have shown that therapeutic induction of ERS-induced apoptosis can be useful in eradicating cancer cells if there is forced activation of ERS (Di et al., 2015; Fan et al., 2016). Recently, the role of ERS signaling in the anticancer activity of icariin has been studied on human adenocarcinoma and esophageal cancer cells. Icariin treatment upregulated ERS-related molecules such as p-PERK, ATF, GRP78, p-eIF2α, CHOP as well as apoptosis-related protein PUMA. At the same time, there was downregulation of anti-apoptosis-related protein Bcl2. This demonstrated that the apoptotic activity of icariin in cancer cells might also be due to activation of ERS signaling (Di et al., 2015; Fan et al., 2016).

Lysosomes are acidic organelles containing various hydrolytic enzymes for recycling of intracellular proteins. When the alteration in lysosomal membrane permeabilization occurred, there will be release of its hydrolytic enzymes, leading to a change in mitochondrial membrane potential and subsequent pro-apoptotic events of cells (Geng et al., 2015). In a recent study, it was shown that treatment with icariside II induced both mitochondrial and lysosomal dysfunction through mitochondrial membrane and lysosomal membrane permeabilization in human hepatoblastoma cells, leading to caspase activation and cell apoptosis (Geng et al., 2015). This shows that icariside II is able to induce cancer cell apoptosis by causing both mitochondrial and lysosomal damage.

**Figure 2:**
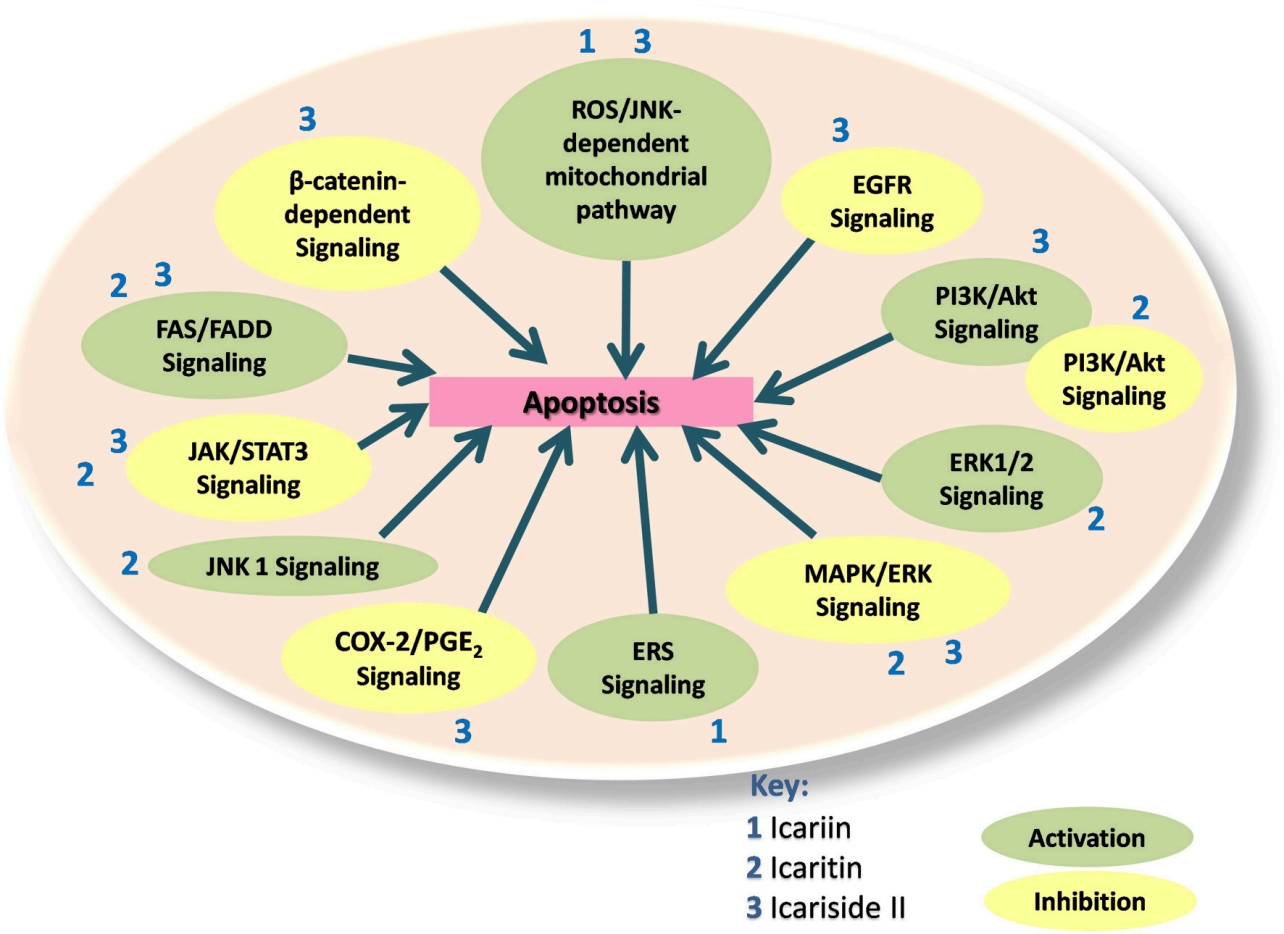
**Icariin, icaritin, and icariside II exert apoptotic effects through multiple mechanisms, which include the inhibition of β-catenin-dependent signaling, EGFR signaling, MAPK/ERK signaling, PI3K/Akt signaling, JAK/STAT3 signaling, and COX-2/PGE_**2**_ signaling**. The activated pathways are such as ROS/JNK dependent mitochondria pathway, FAS-dependent apoptosis, JNK1 signaling, ERS signaling and ERK1/2 signaling. PI3K/Akt signaling can also be activated to inactivate JAK/STAT pathway.

Therefore, icariin and its derivatives appear to be effective in inducing apoptosis in a wide range of cancer cell types through a variety of cell signaling mechanisms (Figure 2).

### Cell-cycle modulation

The cell cycle consists of phases known as G_0_, G_1_, S, and G_2_M. Cyclin-dependent kinases (CDK) are the protein translating signals that push the cells through the cell cycle and the growth of cells is dependent on their capability to translate these signals for effective replication and division. When the normal regulation of cell-cycle progression and division is disrupted, uncontrolled proliferation can occur which may result in the development of cancer (Crespo-Ortiz and Wei, 2012; Dinicola et al., 2014).

**Figure 3:**
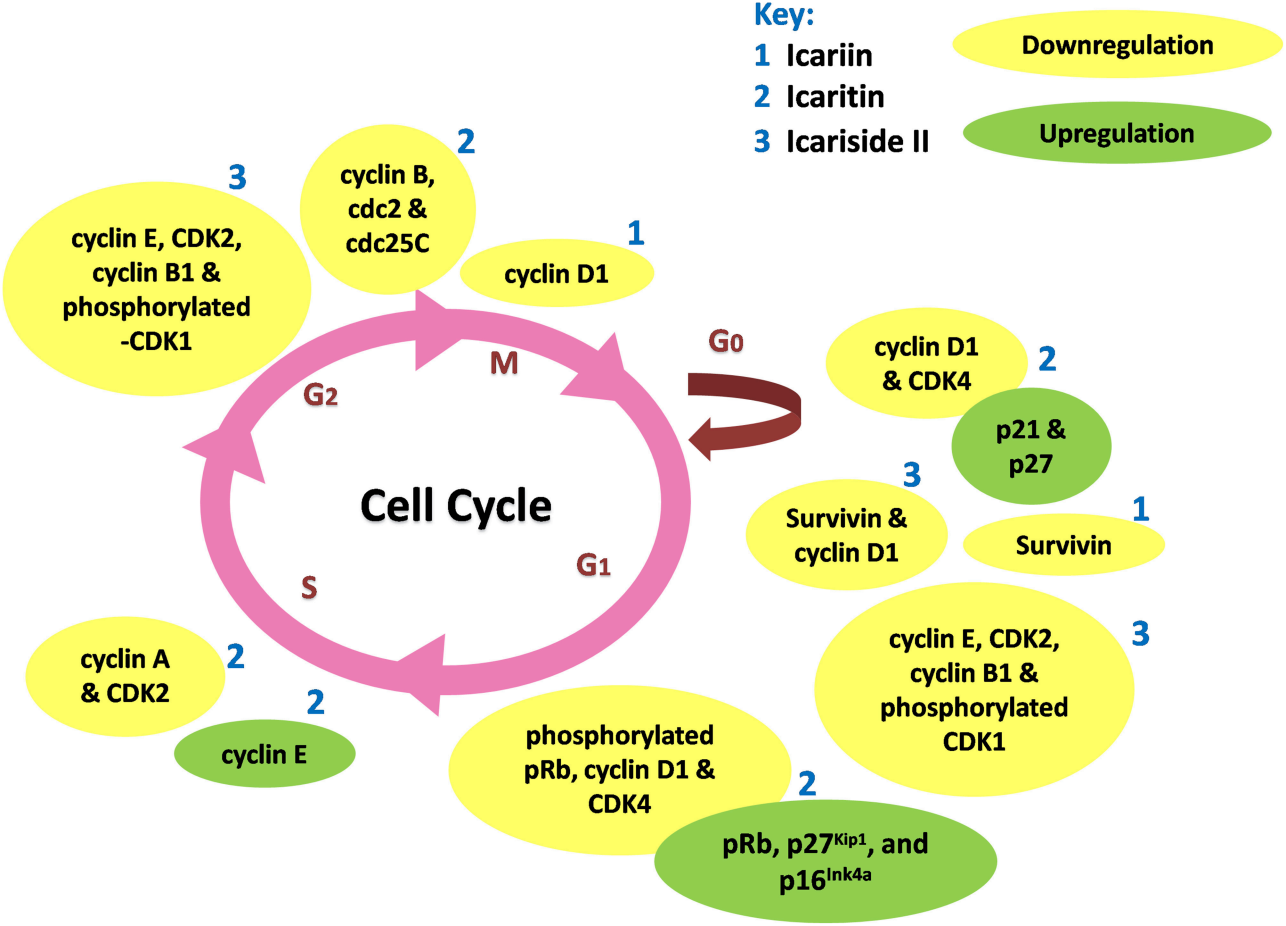
**Cell cycle modulation by icariin and its derivatives**. Icariin, icaritin, and icariside II induce cell cycle arrest through modulation of expression of cell cycle regulatory proteins at different stages of cell cycle, resulted in inhibition of tumor growth.

Icariin and its derivatives inhibit the growth of cancer cells by causing cell cycle arrest. Icariin induces cell cycle arrest at G_0_/G_1_ and G_2_/M phases in gallbladder carcinoma cells and colorectal cancer cells respectively, through the inhibition of NF-κB activity (Zhang et al., 2013, 2014). Icaritin inhibits growth of human prostate carcinoma cells through G_1_ cell cycle arrest which is attributed to induction of p27^Kip1^, p16^Ink4a^, and pRb, as well as the down-regulation of expression of phosphorylated pRb, cyclin D1 and CDK4 (Huang et al., 2007). In other work, icaritin caused cell cycle arrest at the G_2_/M phase in extranodal NK/T-cell lymphoma cells (Wu et al., 2015b). Similarly, treatment of breast cancer cells with icaritin resulted in cell cycle arrest at the G_2_/M phase, accompanied by down-regulation of the expression of G_2_/M phase regulatory proteins including cyclin B, cdc2 as well as cdc25c (Guo et al., 2011). The cell cycle arrest was associated with sustained extracellular signal-regulated kinases (ERK) activation, consistent with the findings of Tong et al. (2011). Aside from these, icaritin has been shown to induce cell cycle arrest in the S phase associated with down-regulated expression levels of S regulatory proteins including Cyclin A, Cyclin B, and CDK2 in lung cancer and chronic myeloid leukemia cells (Zheng et al., 2014; Zhu et al., 2015). As shown in Figure 3, the cell cycle modulation of icaritin seems to vary from study to study and this might be due to the cell type species-differences of the cancer cell lines (Zheng et al., 2014). Icariside II also inhibits cancer cell proliferation through cell cycle modulation (Wang et al., 2011b); it was able to induce cell cycle arrest at G_0_/G_1_ and G_2_/M transitions. The underlying mechanism was suggested to be due to the generation of reactive oxygen species as well as activation of p38 and p53 (Wu et al., 2015a). In a study on osteosarcoma, the inhibition of cancer cell proliferation by icariside II was associated with its ability to inactivate epidermal growth factor receptor (EGFR)/mammalian target of rapamycin (mTOR) pathway, showing that icariside II might target mTOR, the master switch of tumor cell proliferation (Geng et al., 2014).

Based on these studies, icariin and its derivatives were demonstrated to exert cell cycle modulation at different phases resulting in an anti-proliferative effect on cancer cells; mainly attributed to the downregulation of cell cycle regulatory proteins, as summarized in Table 2.

**Table 2:** **Cell cycle modulation of icariin and its derivatives**.

**Compound**	**Cell Line**	**Study Type**	**Mechanism**	**References**
Icariin	Human gallbladder carcinoma cells (GBC-SD and SGC-996)	*In vitro*	G0/G1 phase cell cycle arrest due to inhibition of expression of survivin	Zhang et al., 2013
	Colorectal Cancer Cells (HCT116 and HT29)	*In vitro*	G2/M phase cell cycle arrest through suppression of NF-κB activation and downregulation of Cyclin D1	Zhang et al., 2014
Icaritin	Breast cancer cells (MDA-MB-453 and MCF7)	*In vitro*	G2/M phase cell cycle arrest accompanied by downregulation of the expression levels of the G2/M regulatory proteins such as Cyclin B, cdc2 and cdc25C	Guo et al., 2011
	Human prostate carcinoma cells (PC-3)	*In vitro*	G1 cell cycle arrest through increased expression of pRb, p27^Kip1^, and p16^Ink4a^ as well as decreased expression of phosphorylated pRb, Cyclin D1 and CDK4.	Huang et al., 2007
	Human endometrial cancer cells (Hec1A)	*In vitro*	Reductions of cyclin D1 and cdk4 protein expression and inductions of p21 and p27 expression.	Tong et al., 2011
	Extranodal NK/T-cell lymphoma cells (SNK-10 and SNT-8)	*In vitro*	G2/M phase cell cycle arrest through inhibition of Jak/Stat3 and PI3K/Akt signaling pathway mediated by reduced expression of LMP1.	Wu et al., 2015b
	Human lung cancer cells (A549)	*In vitro*	S phase cell cycle arrest through downregulation of expression levels of S regulatory proteins such as Cyclin A and CDK2	Zheng et al., 2014
	Human multiple myeloma cells U266 (ATCC TIB-196) and Human primary multiple myeloma cells	*In vitro* and *In vivo*	S phase cell cycle arrest downregulation of expression levels of S regulatory proteins such as Cyclin A, Cyclin B and CDK2 and upregulated the expression of Cyclin E	Zhu et al., 2015
Icariside II	Human osteosarcoma cells (MG-63 and Saos-2)	*In vitro* and *In vivo*	Inhibition of epidermal growth factor (EGF)-induced activation of EGFR/mTOR signaling pathway, including EGFR, PI3K/AKT/PRAS40, Raf/MEK/ERK as well as mTOR.	Geng et al., 2014
	Human eosophageal squamous carcinoma (Eca109)	*In vitro* and *In vivo*	Downregulation of survivin and Cyclin D1, inhibition of β-catenin-dependent signaling.	Wang et al., 2011b
	Human melanoma cells (A375)	*In vitro*	G0/G1 and G2/M phases cell cycle arrest through generation of ROS and activation of p38 and p53, accompanied by Inhibited the expression of cell-cycle related proteins, including Cyclin E, CDK2, Cyclin B1, and P-CDK1	Wu et al., 2015a

### Angiogenesis inhibition

Angiogenesis is the vital physiological process of formation of new blood vessels; it is a normal process in growth and granulation tissue formation. Angiogenesis is crucial for cancer development because blood vessels supply the nutrition and transfer channels at every stage of the growth of tumor (Zhang, 2014). Therefore, disruption of angiogenesis can cause inhibition of tumor invasion and metastasis (Tan et al., 2016). Currently, anti-angiogenesis treatment represents one of the strategies in cancer therapy but the currently known effective anti-angiogenesis agents are prohibitively expensive.

**Figure 4:**
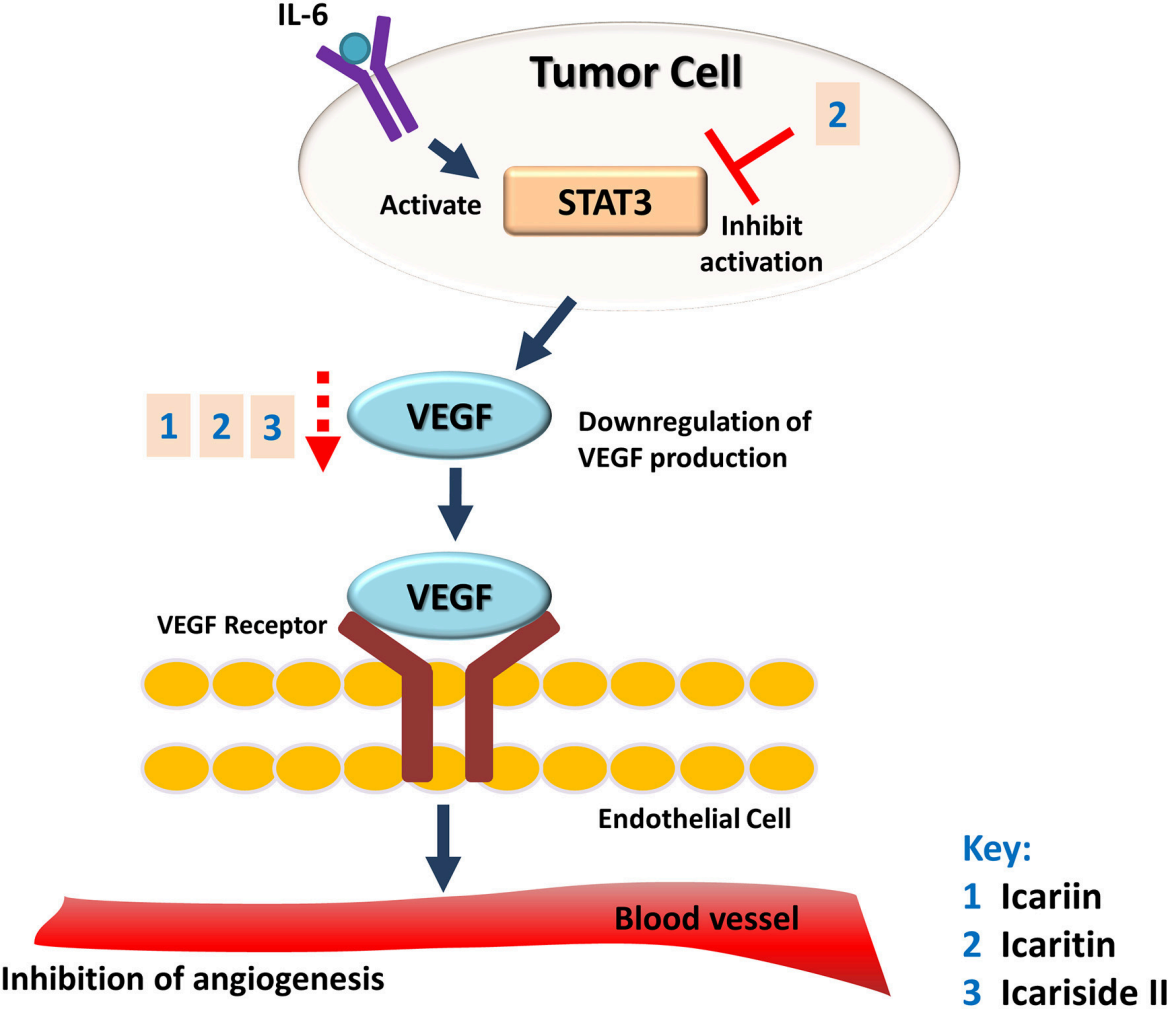
**Anti-angiogenic effect of icariin and its derivatives**. The treatment of icariin, icaritin, and icariside II have resulted in anti-angiogenic effect. This can be due to the reduction in expression of VEGF, a crucial growth factor that acts as the fundamental regulator of angiogenesis.

The potential of *Herba Epimedii* to inhibit angiogenesis was investigated *in vitro* and *in vivo*, as summarized in Table 3. Anti-angiogenic effects of the ethyl acetate fraction of *Herba Epimedii* extract was demonstrated in both experimental models and might be due to inhibition of ERK signaling pathway (Yu et al., 2013). Preliminary studies on the effect of active compounds of *Herba Epimedii* such as icariin and icaritin on angiogenesis have been carried out by using human umbilical vein endothelial cells as a cell model of angiogenesis. Both icariin and icaritin were found to inhibit the proliferation, migration as well as tube formation of the cells (Zhang, 2014; Ye et al., 2015). In a chick embryo chorioallantoic assay, there was significant reduction in newly-formed blood vessels as well as reduced area and length of blood vessels that occurred in a dose-dependent manner following treatment of icariin and icaritin (Hong et al., 2013; Ye et al., 2015). Icariin and its derivatives have also exhibited their anti-angiogenic activity *in vivo* in different xenograft models of tumors such as hepatocellular carcinoma and renal cell carcinoma. CD31 is a pan-endothelial marker which is specifically expressed on the endothelial cells surfaces; in tumors of mice treated with icariin and icaritin, there was a significant decrease in the areas positive for CD31 (Yang et al., 2009; Li et al., 2013b). The anti-angiogenic effect appears to be moderated by down-regulation of vascular endothelial growth factor (VEGF), a crucial growth factor that acts as the fundamental regulator of angiogenesis (Figure 4). The results of both *in vitro* and *in vivo* studies showed a decrease in VEGF levels in both the icaritin-and icariside-II treated groups (Choi et al., 2008; Li et al., 2013b). Based on the study reported by Choi et al. (2008), the decreased level of VEGF is probably due to the reduction in transcriptional activity of hypoxia inducible factor 1-alpha, the primary regulator of the expression of VEGF. Taking all of the above into account, inhibition of VEGF is likely to be the target of icariin and its derivatives with respect their anti-angiogenic effect.

**Table 3:** **Anti-angiogenic effect of icariin and its derivatives**.

**Compound**	**Model/Cell Line**	**Study Type**	**Observation/Mechanism**	**References**
*Herba Epimedii* ethyl acetate fraction	HUVECS and Zebrafish embryos	*In vitro* and *In vivo*	Inhibition of HUVECs proliferation, migration, and VEGF-induced tube formation as well as significant inhibition of blood vessel formation in zebrafish embryos, due to inhibition of ERK signaling pathways	Yu et al., 2013
Icariin	HepG2 tumor	*In vivo*	Decrease in CD31+ vessels in tumor	Yang et al., 2009
	HUVECS and CAM Assay	*In vitro* and *In vivo*	Inhibition of proliferation of HUVECs and inhibition of angiogenesis in CAM, reduced expression of VEGF	Ye et al., 2015
Icaritin	CAM Assay	*In vivo*	Inhibition of angiogenesis in CAM	Hong et al., 2013
	Renca tumor	*In vivo*	Decrease in the mean positive area of CD31 in immunohistochemical analyses of tumor, reduced expression of VEGF, due to inhibition of activity of STAT3	Li et al., 2013b
	HUVECS	*In vitro*	Inhibition of HUVEC proliferation, migration and tube formation	Zhang, 2014
Icariside II	HUVECS	*In vitro*	Inhibition of angiogenic function with reduced widths and lengths of the endothelial network-like structures in HUVECS, reduced expression of VEGF in human osteosarcoma cells	Choi et al., 2008

### Inhibition of metastasis

The malignancy of cancers is mainly due to the ability of cancer cells to invade their neighboring tissues. This process of invasion allows neoplastic cells to enter the blood stream and lymphatic system and reach other organs where secondary tumors may form. The development of metastases is the cause of treatment failure and death in cancer patients. The key steps for metastases are cell attachment, local proteolysis and cell migration (Harlozinska, 2005). Icariin and its derivatives have also been investigated with reference to their anti-metastatic activity. A study on highly metastatic human lung main cancer cells demonstrated a reduced ability of these cells to invade and migrate following treatment with icariin (Mao et al., 2000). In another study, icariin suppressed the adhesion of lung adenocarcinoma (Di et al., 2015). The possible mode of action includes effects on vasodilator-stimulated phosphoprotein (VASP) which is reported to be crucial in cell migration as well tumor metastasis. VASP function in turn is regulated by Rac, a member of the Rho family proteins (Schlegel et al., 2000). The study has also revealed the inhibition of human gastric cancer cell invasion and cell migration by icariin and the underlying mechanism was suggested to be the suppression of expression of cell motility-related genes Rac1 and VASP (Wang et al., 2010).

Icaritin was also tested on glioblastoma cell line. There was suppression of adhesion, migration and invasion of glioblastoma cells, due to downregulation of extracellular matrix metalloproteinase via PTEN/Akt/HIF-1α pathway (Xu et al., 2015). Icariside II was also found to exert anti-metastatic effects as it inhibited the migration of human osteosarcoma cells by suppressing the transcription of hypoxia-inducible genes involved in tumor cell invasion, including urokinase plasminogen activator receptor, adrenomedullin, and matrix metalloproteinase 2 (Choi et al., 2008). The CXCR4/CXCL12 signaling axis is known to be involved in the metastasis of cancer cells and CXCR4 chemokine receptors are highly expressed in various tumors compared to normal cells (Kim and Park, 2014). In a study, the anti-metastatic activity of icariside II was shown to be correlated with downregulation of CXCR4 at transcriptional level through the suppression of NF-κB activation (Kim and Park, 2014). The chronic inflammation that occurs during cancer treatment is an important factor that induces tumor metastasis because an inflammatory microenvironment is favorable for cancer invasion and metastasis through epithelial-mesenchymal transition (EMT). Studies have been done on the effect of exposure to icariside II on the migration of non-small cell lung cancer cells in an inflammatory microenvironment; the exposure of icariside II resulted in the inhibition of TNF-α-boosted EMT, migration and invasion of cancer cells, probably through the inhibition of Akt/ NF-κB pathway. This is supported by the results of an *in vivo* study that showed anti-metastastic effect of icariside II on tumor in nude mice (Song et al., 2016).

**Figure 5:**
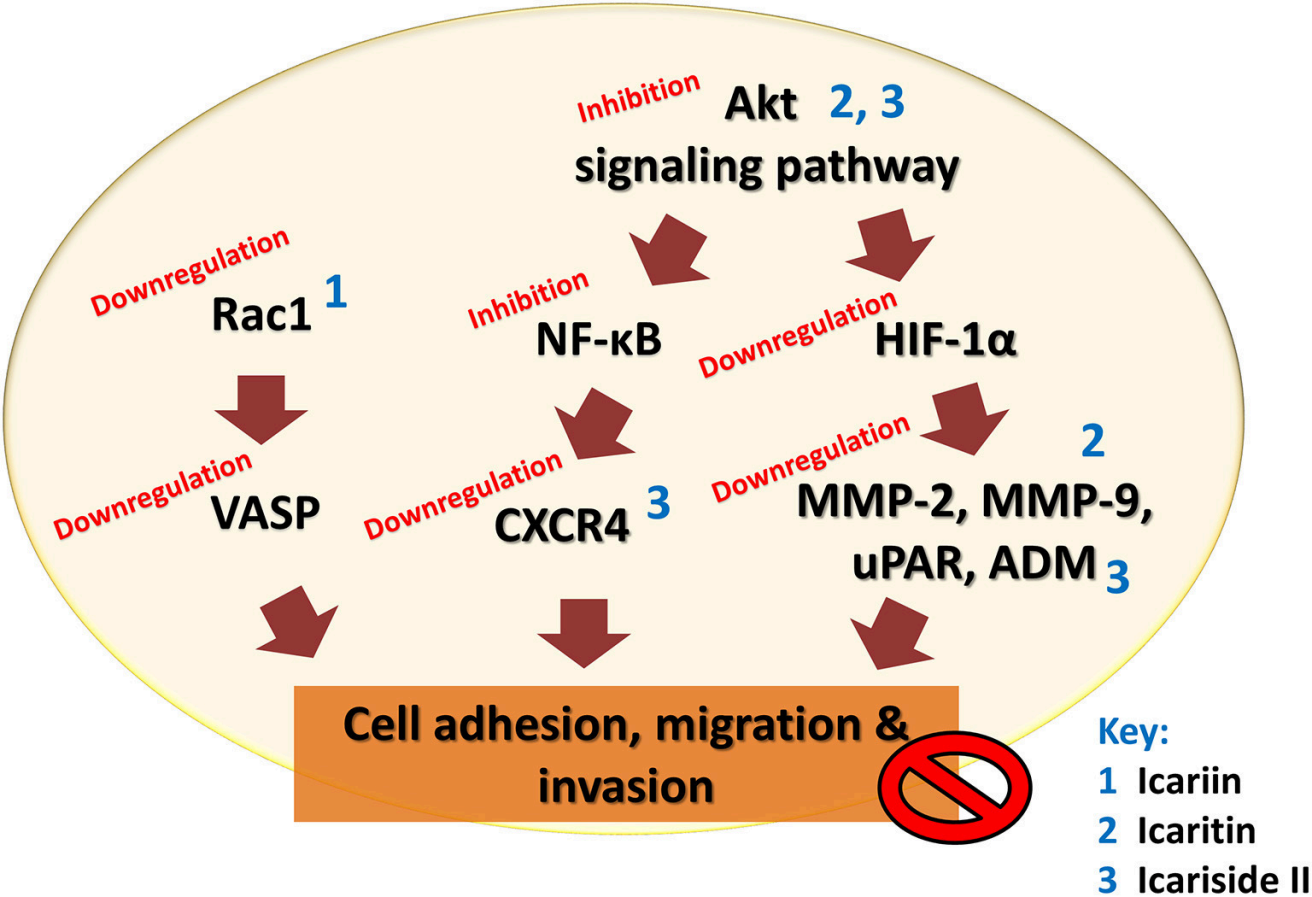
**Anti-metastatic effect of icariin and its derivatives**. Icariin, icaritin, and icariside II inhibit metastasis of tumor by inhibiting cell adhesion, migration, and invasion through multiple mechanisms that resulted in downregulation of VASP, CXCR4, uPAR, ADM, and matrix metalloproteinases.

The findings of these studies revealed that icariin and its derivatives inhibit the metastasis of cancer cells through downregulation of proteins that are crucial for cancer metastasis, as summarized in Table 4 and Figure 5.

**Table 4:** **Anti-metastatic effect of icariin and its derivatives**.

**Compound**	**Cell Line**	**Study Type**	**Mechanism**	**References**
Icariin	Human lung adenocarcinoma cells (A459)	*In vitro*	Decreased migration and adhesion of cells	Di et al., 2015
	Human lung cancer cells (PG)	*In vitro*	Decreased cell adhesive ratio to laminin substrate and reduced cell ability of invasion or migration	Mao et al., 2000
	Gastric carcinoma cells (BGC-823)	*In vitro*	Inhibition of cells migration through downregulation of cell motility-related genes Rac1 and VASP	Wang et al., 2010
Icaritin	Human glioblastoma cells (U87MG)	*In vitro*	Downregulation of EMMPRIN via the PTEN/Akt/HIF-1α signaling pathway	Xu et al., 2015
	Human osteosarcoma cells (SaOS2)	*In vitro*	Decreased cell motility, through downregulation of MMP-2 and MMP-9 expression	Wang and Wang, 2014
Icariside II	Human osteosarcoma cells (HOS)	*In vitro*	Inhibition of the transcription of hypoxia-inducible genes involved in invasion/migration, such as uPAR, ADM, and MMP2	Choi et al., 2008
	Human cervical cancer cells (HeLa) and Breast cancer cells (MDA-MB-231)	*In vitro*	Downregulation of the expression of CXCR4, through suppression of NF-κB activation	Kim and Park, 2014
	Human non-small cell lung cancer cells (A549 and H1299)	*In vitro* and *In vivo*	Inhibit of TNF-α-induced migration and EMT of cancer cells *in vitro* and *in vivo*, through inhibition of Akt/NF-κB pathway	Song et al., 2016

### Inhibition of hormone dependent cancers

Breast and prostate cancers are the commonest invasive hormone dependent cancers in females and males, respectively. Although these two types of cancers have different physiological features depending on the organs where they arise, they have common pathological features of “hormonal-dependence” (Belev and Vrbanec, 2013). The initiation and progression of these diseases are based on the modulation of hormone effects through specific steroid hormone receptors including estrogen receptors, progesterone receptors and androgen receptors (Rau et al., 2005).

Estrogens are known to stimulate the growth of breast cancer and most breast cancers have overexpression of estrogen receptor alpha (ERα). It has been found that some ERα –positive tumors can develop resistance toward hormone therapy. These indicate a role of ERα in initiation and progression of cancer (Ali and Coombes, 2000). Icaritin has been reported to inhibit the estradiol-stimulated growth of ERα-positive breast cancer cells by destabilizing ERα protein. Icaritin activates the aryl hydrocarbon (Ahr)-mediated pathways by acting as an Ahr agonist; resulting in Ahr-mediated proteosomal pathways and degradation of ERα protein which ultimately reduces the maximal estrogen responsiveness. In an athymic nude mouse model, icaritin also restricted estradiol-stimulated breast cancer xenograft growth and caused strong reduction in ERα protein levels (Tiong et al., 2012). This finding is consistent with another recent study which showed undetectable levels of ERα protein in breast cancer tumors of nude mice due to the presence of icaritin and desmethylicaritin in serum after the administration of *Epimedium bervicornum* extract (Indran et al., 2014).

Androgens play a crucial role for survival and growth of prostatic carcinoma cells; hence, androgen deprivation therapy has been used as the mainstay of treatment for prostate cancer. This includes the reduction of gonadal androgen synthesis as well as disruption of androgen receptor (AR) signaling axis. However, the mechanism of cancer development has evolved and some variants have developed into castration-resistant prostate cancer (CRPC) (Belev and Vrbanec, 2013). This occurs where there is persistent AR signaling which is postulated to be produced by AR COOH-terminal truncated splice variants (ARvs) (Sun et al., 2015a). Recently, icaritin has been tested on CRPC cells and was found to exert similar effects to those seen in estrogen responsive breast cancer, with promotion of degradation of AR and ARvs through the binding of icaritin to Ahr. Consequently, the transcription of genes regulated by AR and ARvs such as *KLK3* and *UBE2C* are suppressed, inducing apoptosis of cancer cells. In an *in vivo* study, reduction of tumor growth was seen in murine models implanted with androgen-sensitive and CRPC cells, due to inhibition of AR signaling by icaritin (Sun et al., 2015a). In another study, the treatment of androgen receptor-positive prostate cancer cells with icariside II resulted in downregulation of expression of *KLK3* gene, showing the role of icariside II in inhibition of AR signaling (Miura et al., 2015).

Based on the research, icaritin and its derivative are effective against hormone dependent cancers that are resistant toward hormone therapy by promoting the degradation of hormone receptors through Ahr-dependent pathway.

### Inhibition of cancer stem cells

Cancer stem cells (CSCs), also known as cancer/tumor-initiating cells, can be defined as a small population of cancer cells that are able to self-renew and differentiate into a wide range of progeny cells that make up the tumor as well as reform the original tumor following xenotransplantation (Kim et al., 2009). Although conventional cancer therapy—including radio and chemotherapy—destroy the bulk of cancer cells, they often fail to eradicate the critical CSCs due to inherent resistance mechanisms. Consequently, CSCs form new tumors, which results in relapse of the cancers (Vinogradov and Wei, 2012). The inhibitory effect of icaritin on CSCs has been evaluated with one study reporting more effective inhibition of growth of breast cancer stem/progenitor cells following treatment with icaritin compared to the standard chemopreventive agent tamoxifen (Guo et al., 2011). Recently, the inhibition of malignant growth of hepatocellular carcinoma initiating cells (HCICs) by icaritin has been demonstrated (Zhao et al., 2015). The hepatosphere cells (HCIC that formed spherical clusters) were inhibited from forming secondary passage of hepatospheres due to the effect of icaritin which negatively regulated the self-renewal of HCICs. In addition, the hepatosphere cells lost their ability to initiate tumor. In *in vivo* work, growth of primary and secondary xenografts in NOD/SCID mice were also suppressed. The underlying mechanism for these observed effects was proposed to be the attenuation of IL-6 receptors/Jak2/Stat3 signaling (Zhao et al., 2015). These recent findings show the potential of icaritin in eradicating CSCs; and seem to suggest that it may be worthwhile conducting further studies on icariin and its other derivatives to evaluate their effectiveness against other types of CSCs.

### Inhibition of resistant cancer cells

Another huge challenge for anticancer therapy is the emergence of drug resistance over the course of treatment which has led to drug-limiting toxicities and failure of chemotherapy (Crespo-Ortiz and Wei, 2012; Chan et al., 2016). The resistance of cancer cells can develop via biological mechanisms such as reduced uptake of drugs, changes in cellular pathways and increased drug efflux. The most common mechanism is overexpression of ATP-binding cassette (ABC) transporters such as P-glycoprotein (P-gp) that extrudes the chemotherapeutic agents from the cells (Liu et al., 2009). In the study by Song et al. (2014), the combined treatment of *Epimedium koreanum* Nakai extract with gefitinib overcame the resistance of non-small cell lung cancer cells to gefitinib, showing the potential of bioactive components in *Herba epimedii* to treat cancers with drug-resistance. Hence, studies have been carried out to unravel the reversal of drug resistance of tumor cells by icariin and its derivatives. One study using multiple drug-resistant (MDR) human hepatocellular carcinoma/Adriamycin (ADR) cell line revealed the capability of icaritin to reverse the cytotoxicity of ADR in a dose-dependent manner, which was due to the decrease in expression of MDR1 gene, decrease in P-gp level and increased intracellular accumulation of ADR (Sun et al., 2013). Similar results were obtained in a study on MDR reversal activities of a series of semi-synthesized icariin derivatives using ADR-resistant breast carcinoma cells (Liu et al., 2009). In another study, the treatment of icariin resulted in the inhibition of the P-gp-mediated active transport process. In addition, there was enhanced doxorubicin (DOX)-induced apoptosis in DOX-resistant human osteosarcoma cells and this was due to the inhibition of PI3K activity and reduced phosphorylation of Akt in the cells (Wang et al., 2015b). Recently, icaritin has been shown to overcome the resistance of human glioblastoma cells to apoptosis caused by tumor necrosis factor-related apoptosis-inducing ligand (TRAIL). Treatment with icaritin has been shown to cause the inhibition of NF-κB signaling which in turn leads to the suppression of the expression of cellular PLICE-inhibitory protein (c-FLIP), the inhibitor of apoptotic signaling. Consequently, TRAIL-induced apoptosis in glioblastoma cells can be sensitized (Han et al., 2015). Overall, icariin and its derivatives are able to reverse the drug resistance of cancer cells, mainly by inhibiting the activity of P-gp and promoting the accumulation of drug in cancer cells.

### Immunomodulation

There is increased interest in focusing the application of tumor immunology for cancer therapy (Ménard et al., 2008). Myeloid-derived suppressor cells (MDSCs) are part of the myeloid-cell lineage and they are the heterogenous population of cells which consist of myeloid-cell progenitors and precursors of myeloid cells. In healthy individuals, MDSCs quickly differentiate into mature granulocytes, macrophages, or dendritic cells (DCs). However, in pathological conditions such as cancer, differentiation of MDSCs and subsequent expansion of the cell population is blocked (Gabrilovich and Nagaraj, 2009). As MDSCs possess immunosuppressive functions, their accumulation is thought to be crucial in protecting the tumor cells from immune-mediated killing which then facilitates tumor growth and metastasis, especially in solid cancers. Therefore, targeting MDSCs could be another approach for cancer therapy (Marvel and Gabriolovich, 2015). In a study, *in vitro* treatment of MDSCs with icaritin led to a reduced proportion of MDSCs with induction of differentiation of the cells into macrophages and DCs. There have been *in vivo* experiments with tumor bearing mice where treatment with icaritin and icariin has delayed tumor progression and reduced the percentage of MDSCs. The levels of arginase, nitric oxide and ROS were determined as they are involved in MDSC-mediated T-cell suppression and the results showed significantly lower production of nitric oxide and ROS as well as restoration of CD8^+^ T cells functional capacity. In addition to that, there was downregulation of expression of S100A8 and S100A9, the pro-inflammatory mediators that inhibit the differentiation of MDSCs. There was also inhibition of activation of STAT and AKT (Zhou et al., 2011). Based on these studies, the immunomodulatory effect of icariin and icaritin indicates that they are not only effective in killing tumor cells but they are also involved in antitumor immunity for tumor regression.

### Applications in medicines

Besides the studies on icariin and its derivatives alone as potential anticancer agents, there is tremendous interest in using these compounds in combination with standard anticancer chemotherapy, as shown in Table 5. This is to enhance the efficacy of the chemotherapy while reducing the side effects and complications (Wang et al., 2015c). While treatment with either icariin or temozolomide resulted in inhibition of cell proliferation of glioblastoma cells, cell apoptosis and cell migration inhibition; a combination of icariin and temozolomide led to significant enhancement of these antitumor activities compared to the treatment using either agent alone. The synergistic effect was correlated with the suppression of NF-κB activity (Yang et al., 2015). This is consistent with synergistic effects that were observed in other *in vitro* and *in vivo* studies using different anticancer drugs such as gemcitabine, 5-florouracil and arsenic trioxide. Other than inhibition of NF-κB activity, an increase in the generation of intracellular ROS was observed (Zhang et al., 2013; Li et al., 2014a; Shi et al., 2014; Wang et al., 2015c). Recently, the effect of icaritin used in combination with epirubicin against bladder cancer cells was investigated. The results showed that icaritin acted synergistically with epirubicin via suppression of autophagy (Pan et al., 2016). For extranodal NK/T-cell lymphoma cells, icaritin potentiated ganciclovir-induced apoptosis through induction of lytic Epstein–Barr virus infection (Wu et al., 2015b). With reference to studies on icariside II, a synergistic effect was also observed in different cell lines when it was used in combination with drugs such as bortezomib, thalidomide and paclitaxel. There was an increase in the expression of cleaved caspase 3, showing that icariside II potentiated the apoptosis of cancer cells induced by these chemotherapeutic agents with the possible mechanisms involved being inhibition of STAT3 signaling pathways as well as TLR4-MyD88-ERK signaling (Kim et al., 2011; Wu et al., 2012).

**Table 5:** **Applications of icariin and its derivatives with current cancer therapy**.

**Compound**	**Cell Line**	**Study Type**	**Drug/Treatment**	**Effect**	**References**
Icariin	Human Hepatocellular Carcinoma (SMMC-7721 and HepG2)	*In vitro* and *In vivo*	Arsenic trioxide	Potentiates antitumor effect of arsenic trioxide through by generation of intracellular ROS and inhibition of NF-κB activity	Li et al., 2014a
	Human colorectal carcinoma cells (HT29 and HCT116)	*In vitro* and *In vivo*	5-fluorouracil	Potentiates antitumor effect of 5-fluorouracil through inhibition of NF-κB activity	Shi et al., 2014
	Human myeloid leukemia cells (HL-60 and NB4)	*In vitro*	Arsenic trioxide	Potentiates antitumor effect of arsenic trioxide through generation of intracellular ROS	Wang et al., 2015c
	Human glioblastoma cells (U87MG)	*In vitro*	Temolozomide	Potentiates antitumor effect of temolozomide through inhibition of NF-κB activity	Yang et al., 2015
	Human gallbladder carcinoma cells (GBC-SD and SGC-996)	*In vitro* and *In vivo*	Gemcitabine	Potentiates antitumor effect of gemcitabine through inhibition of NF-κB activity and downregulation of NF-κB -regulated gene products	Zhang et al., 2013
	Colorectal Cancer Cells (HCT116 and HT29)	*In vitro* and *In vivo*	X-ray irradiation	Potentiate radiation-induced apoptosis of cancer cells through suppression of radiation-induced NF-κB activation and downregulation of NF-κB -regulated gene products	Zhang et al., 2014
Icaritin	Murine breast cancer cells (4T1)	*In vitro*	Ionizing radiation	Potentiate radiation-induced apoptosis and inhibition of cell proliferation as well as suppression of IR-induced survival path, ERK1/2 and AKT	Hong et al., 2013
	Bladder cancer cells (BT5637 and T24)	*In vitro*	Epirubicin	Inhibition of epirubicin-induced autophagy and synergistic effect with epirubicin to suppress cancer cell proliferation	Pan et al., 2016
	Extranodal NK/T-cell lymphoma cells (SNK-10 and SNT-8)	*In vitro*	Ganciclovir	Potentiates ganciclovir-induced apoptosis through induction of lytic EBV infection	Wu et al., 2015b
Icariside II	Multiple myeloma cells (U266)	*In vitro*	Bortezomib and Thalidomide	Enhanced cytotoxicity of bortezomib and thalidomide through enhancement of apoptosis	Kim et al., 2011
	Human melanoma cells (A375)	*In vitro*	Paclitaxel	Potentiates paclitaxel-induced apoptosis through inhibition of paclitaxel activated TLR4–MyD88–ERK signaling	Wu et al., 2012

Other than chemotherapy, ionizing radiation (IR) therapy is also used as a main treatment of many types of malignancies. However, there are some drawbacks of IR therapy such as treatment-induced resistance, epidermitis and higher risk of cardiovascular disease. Hence, there is a need for an agent or radiosensitizer that can overcome radioresistance and sensitize the malignant cells to radiation. This will also help to enhance the tumor response facilitating the use of lower therapeutic doses of radiation which will help reduce toxicity to other organs (Hong et al., 2013; Zhang et al., 2014). The synergistic effect of icaritin with IR has been demonstrated (Hong et al., 2013) through three main pathways. Firstly, icaritin blocks the activation of survival signaling such as ERK1/2 and AKT pathways of breast cancer cells that are caused by IR stress. Secondly, icaritin enhances the accumulation of cells in G_2_/M phase, causing more cells to die. Thirdly, the induction of necrosis and apoptosis of cancer cells by icaritin occurs in a dose-dependent manner, preventing the IR-insulted cells from repairing the damage. Similar results were obtained in a study that used icariin as a radiosensitizer of colorectal cancer cells (Zhang et al., 2014). One of the possible underlying mechanisms for these observed effects is the suppression of NF-κB activity by icaritin, which then prevents the activation of pro-proliferative genes such as ERK1/2 and AKT as well as causing cell cycle arrest in G_2_/M phase. Based on the reported studies, co-treatment using icariin and its derivatives in combination with standard cancer therapies results in positive effects because they potentiate apoptosis of cancer cells induced by chemotherapy through their action on different signaling pathways. In addition to that, they also help to overcome the radioresistance of cancer cells.

There are a number of patents registered related to the application of icariin and its derivatives in cancer treatment. For example, icariin has been used to treat melanoma and breast cancers (Dong et al., 2011). Icaritin has been utilized in the preparation of medicaments for primary liver cancer and inhibition of liver cancer stem cells (Meng et al., 2014, 2015); while Zhang and Calderwood (2008) reported the application of desmethylicaritin as the active ingredient of an antitumor drug. Broad-spectrum antitumor effects have been shown and it is found to be maintained at a particular concentration in plasma, which is a desirable property for antitumor effect. Icaritin has also been used together with juglanin B for the treatment of hepatocellular carcinoma (Zhao et al., 2014). Other derivatives such as icariinaglycon and cyclo-icariin have also been utilized in treatment of different cancer cell lines (Wang et al., 2011c; Jia et al., 2014). The preparations of icariin and its derivatives for cancer treatment appear to be advantageous because they show low toxicity to normal cells and no obvious adverse effects (Meng et al., 2014; Wei et al., 2015).

### Comparison of potency of icariin derivatives and challenges in clinical usage

In a comparative study done by Huang et al. (2007), icariin and icaritin exert anticancer effect against PC-3 cells to different extents. Icaritin had a stronger effect on PC-3 cells by causing G_1_ and G_2_/M arrest whereas icariin only caused weak G_1_ arrest. The different level of potency of the two compounds in inhibiting cell growth could be due to the difference in their chemical structures. The possible structure-activity relationship was further demonstrated by another study with icariin and icaritin. Although both icariin and icaritin exerted immunomodulatory and antitumor effects, icariin showed weaker action as it did not affect the proportion of MDSCs, macrophages and DCs *in vitro* and furthermore, had no effect on the expression of S100A8/9 in MDSCs *in vitro* (Zhou et al., 2011).

Pharmacological studies have clearly shown that icariin, icaritin and icariside II possess anticancer activity but the challenge for their experimental studies is the small amount of natural icaritin and icariside II present in *Herba Epimedii*. In order to increase the yield of icaritin and icariside II from icariin, enzyme hydrolysis methods have been used to prepare higher amounts of these metabolites for pharmacological studies (Xia et al., 2010; Jin et al., 2012). Another impediment to the clinical application of icariin, icaritin and icariside II is their low solubility in water which will lead to low *in vivo* bioavailability (Cai et al., 2011; Wang et al., 2015a). In order to enhance their water solubility, other derivatives have also been synthesized. For example, Wang et al. (2015a) prepared a series of icaritin derivatives bearing carboxylic acid or carboxylic ester groups and their enhanced cytotoxic activity against breast and lung cancer cells was demonstrated. Song et al. (2015) demonstrated another approach for the enhancement of solubility, which is by incorporating icariside II into phospholipid/d-α-tocopheryl polyetheyene glycol 1000 succinate (TPGS) mixed micelles. The mixed micelles loaded with icariside II showed stronger inhibition action on the proliferation of breast cancer cells compared to icariside II alone. Similar effects were observed in lung cancer cells treated with lecithin/Solutol HS 15 loaded with icariside II (Yan et al., 2015). These results demonstrate that icariin and its derivatives have real potential given that one of the main potential limitations of low solubility can be overcome via the application of novel drug delivery systems.

## Conclusion

**Figure 6:**
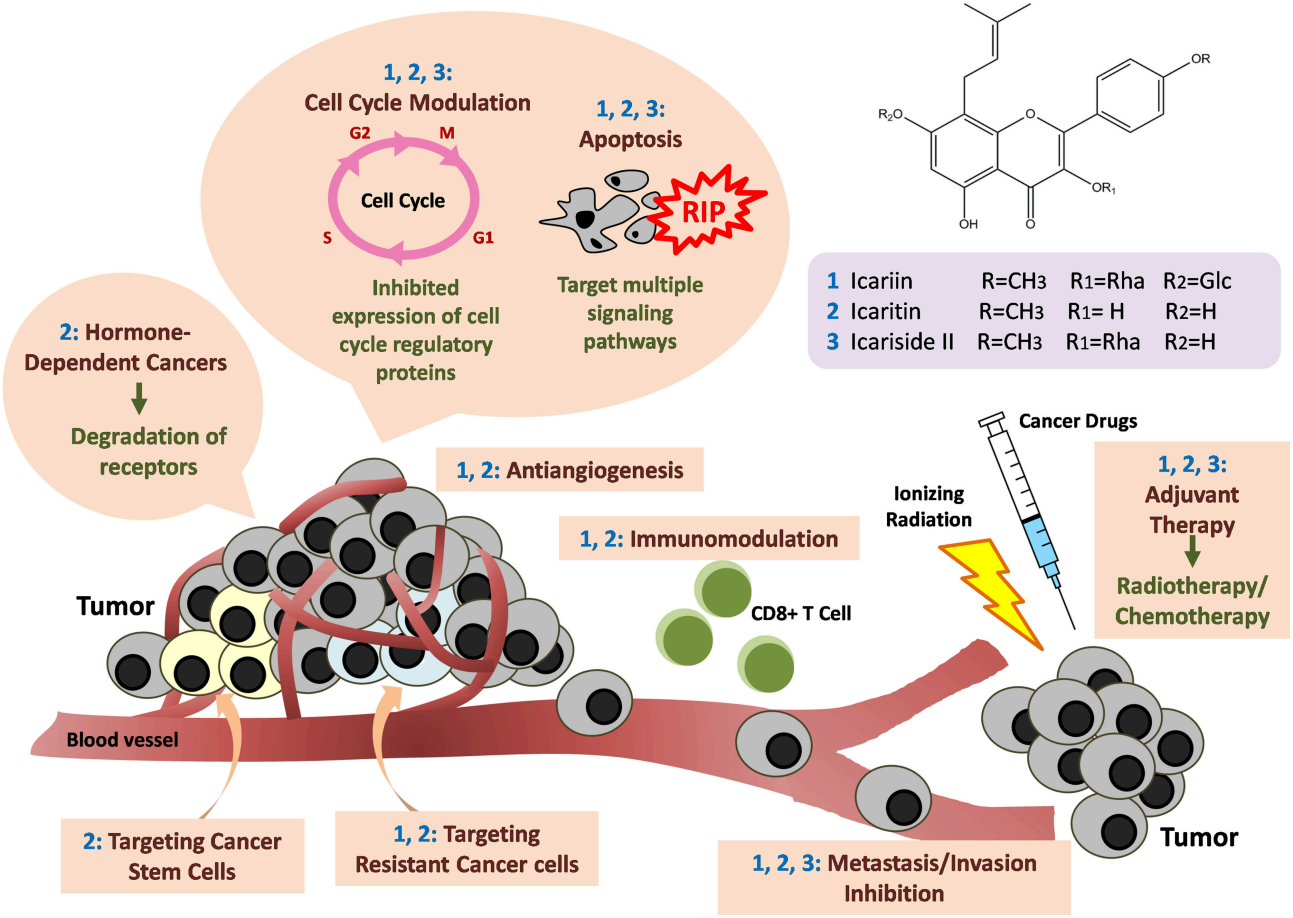
**Anti-cancer mechanisms of icariin and its derivatives**. Icariin and its derivatives mainly inhibit the growth of tumor through induction of apoptosis by targeting multiple signaling pathways. Cell cycle arrests also occur through downregulation of the expression of cell cycle regulatory proteins. Besides, there are anti-angiogenesis, anti-metastasis and immunomodulation. The compounds are also effective in targeting hormone-dependent cancers, cancer stem cells, and drug-resistant cancer cells. There are synergistic effects when icariin and its derivatives are used together with standard cancer therapy such as chemotherapy or radiotherapy.

Overall, the existing research provides supporting scientific-based evidence that substantiates the use of *Herba Epimedii*-containing herbal medicine for anticancer treatment. The active compound in *Herba Epimedii*, icariin and its derivatives possess tremendous potential for treatment of many different cancers—including solid and non-solid tumors. They were found to act through a wide variety of mechanisms as shown in Figure 6. The tantalizing promise of these compounds as suggested by the current work is the ability of these substances to overcome the limitations of current anticancer therapy—including the ability to effectively kill cancer stem cells and help overcome hormone therapy resistance in hormone dependent cancers. Their anticancer activity which goes beyond cytotoxicity to include more novel mechanisms such as immunotherapy is also a fascinating aspect which begs further study. Given that icariin and its various derivatives seem to have different levels of potency with reference to their anticancer effects, work remains to be done to identify the ones with the strongest anticancer activity; this is at the moment challenging as the full details of their mechanisms of action are still not known and the amount of comparative studies at present are limited. The safety profile of these compounds appears promising as clinical trials and a patents study suggest that they can safely be used without adverse effects. All in all, icariin and its derivatives have tremendous potential as candidates for research in the development of new anti-cancer agents.

## Author contributions

All authors listed, have made substantial, direct and intellectual contribution to the work, and approved it for publication.

### Conflict of interest statement

The authors declare that the research was conducted in the absence of any commercial or financial relationships that could be construed as a potential conflict of interest.

## Abbreviations

ADM: adrenomedullin
AIF: Apoptosis inducing factor
Apaf-1: Apoptotic protease activating factor 1
ATF: activating transcription factor
Bad: Bcl-2-associated agonist of cell death
Bax: Bcl-2-associated X protein
Bcl: B-cell lymphoma 2
CAM: Chick *chorioallantoic membrane*
CDK: cyclin-dependent kinases
CHOP: C/EBP homologous protein
COX-2: cyclooxygenase-2
c-src: Proto-oncogene tyrosine-protein kinase Src
CXCR4: chemokine (C-X-C Motif) receptor 4
EBV: *Epstein*-*Barr virus*
eIF2α: α -subunit of eukaryotic translational initiation factor 2
EGFR: Epidermal growth factor receptor
EMMPRIN: extracellular matrix metalloproteinase
EMT: *epithelial*-*mesenchymal transition*
ERK: extracellular signal-regulated kinases
ERS: endoplasmic reticulum stress
FADD: *Fas*-associated protein with death domain
GRP: glucose regulated protein
HIF-1α: hypoxia-inducible factor 1-alpha
HUVECs: human umbilical vein endothelial cells
iNOS: Inducible nitric oxide synthase
IR: Ionizing radiation
JNK: Jun N-terminal kinases
LMP1: Latent membrane protein 1
MAPK: Mitogen-activated protein kinases
MMP: matrix metalloproteinase
mTOR: mechanistic target of rapamycin
NF-κB: nuclear factor-kappa beta
PARP: poly ADP-ribose polymerase
PGE_2_: prostaglandin E2
PI3K: phosphoinositide 3-kinase
Piwil4: piwi-like protein 4
pRb: retinoblastoma protein
PTEN: phosphatase and tensin homolog
PUMA: p53 upregulated modulator of apoptosis
ROS: reactive oxygen species
STAT3: signal transducer and activator of transcription 3
TLR4: toll-like receptor 4
uPAR: urokinase plasminogen activator receptor
VASP: Vasodilator-stimulated phosphoprotein
VEGF: vascular endothelial growth factor.
